# Colorimetric Sugar Sensing Using Boronic Acid-Substituted Azobenzenes

**DOI:** 10.3390/ma7021201

**Published:** 2014-02-14

**Authors:** Yuya Egawa, Ryotaro Miki, Toshinobu Seki

**Affiliations:** Faculty of Pharmaceutical Sciences, Josai University, Keyakidai, Sakado, Saitama 350-0295, Japan; E-Mails: rmiki@josai.ac.jp (R.M.); sekt1042@josai.ac.jp (T.S.)

**Keywords:** azobenzene, boronic acid, glucose sensor, sugar sensor, ^15^N NMR

## Abstract

In association with increasing diabetes prevalence, it is desirable to develop new glucose sensing systems with low cost, ease of use, high stability and good portability. Boronic acid is one of the potential candidates for a future alternative to enzyme-based glucose sensors. Boronic acid derivatives have been widely used for the sugar recognition motif, because boronic acids bind adjacent diols to form cyclic boronate esters. In order to develop colorimetric sugar sensors, boronic acid-conjugated azobenzenes have been synthesized. There are several types of boronic acid azobenzenes, and their characteristics tend to rely on the substitute position of the boronic acid moiety. For example, *o*-substitution of boronic acid to the azo group gives the advantage of a significant color change upon sugar addition. Nitrogen-15 Nuclear Magnetic Resonance (NMR) studies clearly show a signaling mechanism based on the formation and cleavage of the B–N dative bond between boronic acid and azo moieties in the dye. Some boronic acid-substituted azobenzenes were attached to a polymer or utilized for supramolecular chemistry to produce glucose-selective binding, in which two boronic acid moieties cooperatively bind one glucose molecule. In addition, boronic acid-substituted azobenzenes have been applied not only for glucose monitoring, but also for the sensing of glycated hemoglobin and dopamine.

## 1. Introduction

The growing number of people with diabetes is a serious issue worldwide. It has increased from 153 million in 1980 to 347 million in 2008 [1]. The blood glucose level of diabetes patients is abnormally high, and this leads to severe complications, such as cardiovascular disease, chronic renal failure and diabetic retinopathy. Usually, full recovery from diabetes is difficult; however, early treatment of diabetes will prevent complications. Thus, early recognition of diabetes is important. For this purpose, urine test strips have been widely used. They are easy to use, painless and low cost, and there is no need for electronic devices. This would be continuously important, because more than 80% of diabetes deaths occur in low- and middle-income countries [2].

The self-monitoring of blood glucose is recommended for diabetes patients who control their blood glucose level with medicines [3–6]. They monitor their glucose level to ensure their proper medication. In addition, some diabetes patients have to monitor their blood glucose level to adjust their applied dose of insulin injections to avoid hypoglycemia. Widely used enzyme-based glucose sensors can only provide a single datum at the time of measurement. However, the glucose level fluctuates throughout the day. In general, glucose level is lowest in the morning and rises after meals. Accordingly, it is recommended that type 1 diabetes patients, who have defects in insulin production, perform self-blood glucose monitoring at least three times a day [3].

Currently, practical glucose sensing systems have been designed with enzyme reactions. Usually, a test strip contains glucose oxidase, peroxidase and chromogen. For a blood glucose sensing system, glucose oxidase or glucose dehydrogenase is immobilized on the surface of electrodes. The enzymes selectively bind glucose, even in a mixture of sugars, and catalyze the oxidation of glucose. Recently, continuous monitoring systems based on enzymes have been developed and used practically. However, they need improvement with some problems in accuracy, the need for calibration, the invasiveness, the short usable period and the difficulties associated with sterilization [4–6].

In order to compensate for the imperfection of glucose monitoring systems, chemists are trying to replace the enzymes with synthetic chemical ligands, because they are considered to be more stable, easier to handle and low cost compared to enzyme-based systems. Several approaches are ongoing [7–12], and the most promising chemical ligand is boronic acid, which reversibly forms a cyclic ester with the *cis*-1,2- or 1,3-diol structures of sugars. This review provides a brief overview of the sugar binding ability of boronic acids, and the applications of them in optical sensing systems, and summarizes recent studies for a colorimetric sugar sensing system using boronic acid-substituted azobenzene derivatives.

## 2. Binding Ability of Boronic Acids

**Figure 1.**
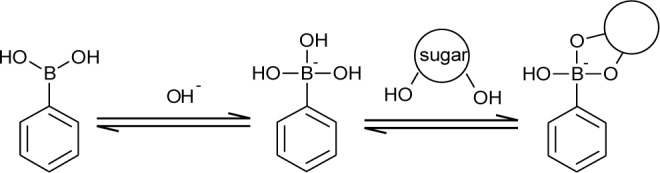
Equilibriums of phenylboronic acid and sugar.

Boronic acid is a kind of Lewis acid. Its sp^2^ boron center has a vacant p orbital that can accept a lone pair of a Lewis base. In aqueous media, boronic acids interact with a hydroxide ion, which results in a conformational change to sp^3^ hybridized boronates. The formation of boronates is dependent on the concentration of the hydroxide ion. In other words, the boronate formation is pH dependent. To express the acidity of boronic acids, an acid dissociation constant, *K*_a_, and a logarithmic constant, p*K*_a_, are usually used. When the pH of the aqueous solution is adjusted to the p*K*_a_ of boronic acids, the concentration of the boronic acid form is equal to the concentration of the boronate form. The acidity of boronic acid tends to conform to Hammett equation-like carboxylic acids [13]. It is widely known that sugar addition induces a decrease of the apparent p*K*_a_. This phenomenon is explained by using a theory demonstrated by Lorand and Edwards [14]. They have concluded that the boronate form plays the major role for binding diol structures compared to the boronic acid form (Figure 1). It is reported that the p*K*_a_ of phenylboronic acid is approximately nine [13]. In neutral or weak alkaline solution, a certain degree of the boronate form exists. Upon sugar addition, the boronate form binds the hydroxyl groups of sugar to form cyclic ester, resulting in the consumption of the boronate form. To maintain the acid-base equilibrium between the boronic acid form and the boronate form, the boronic acid form changes to the boronate form. Accordingly, sugar addition induces the decrease of the boronic acid form, leading to a decrease of apparent p*K*_a_. This structural change upon sugar addition is very important for signaling the mechanism of a chemical sugar sensor based on boronic acids.

It is generally recognized that mono-boronic acid derivatives show higher affinity for D-fructose over D-glucose (Table 1) [15].

James *et al.* pointed out that this is due to the fact that boronic acids prefer the diol of the furanose form [16]. D-Fructose has some configurations, and the relative percentage of the β-D-fructofuranose form is 25% in D_2_O at 27 °C [17]. In contrast, D-glucose shows only 0.14% of α-D-glucofuranose in D_2_O at 31 °C [18]. They proposed that the difference in affinity is strongly associated with this difference of the amount of the furanose form.

A post-meal glucose level of less than 10 mM and a fasting plasma glucose of 3.9 to 7.2 mM [19] are recommended. Table 1 shows that the binding constant of phenylboronic acid to D-glucose is 4.6 M^−1^, which means that the dissociation constant of that is 217 mM. Compared to the blood glucose level, the dissociation constant is too high, which suggests that the practical use of phenylboronic acid for blood glucose monitoring is difficult.

For a stronger binding for D-glucose, bis-boronic acids have been developed in synthetic approaches. In 1995, James *et al.* first reported that a fluorescent sensor containing two boronic acid moieties shows a selective and higher affinity for D-glucose [20,21]. The fluorescent sensor covered the clinical range of the blood glucose level. Norrild *et al.* have demonstrated that all five hydroxyl groups of α-D-glucofuranose in the furanose form are bound by bis-boronic acid [22,23].

It is possible to verify the selectivity to a specific sugar other than glucose by a proper arrangement of two boronic acid groups [24–26]. Furthermore, some bis-boronic acids are able to discriminate the chirality of target molecules [26–29].

## 3. Optical Sugar Sensor Based on Boronic Acid

Glucose oxidase in a blood sugar sensor binds D-glucose and catalyzes its oxidation, which is the trigger of the electrochemical signal. In other words, glucose oxidase serves as both the molecular recognition motif and transducer motif. The dual role of the enzyme is very useful for fabricating sensors. In contrast, boronic acid works as only a molecular recognition motif, and it should be combined with a transducer motif that produces a signal change upon sugar binding. Fluorescent dyes are most widely used as a transducer, because fluorescent dyes provide a signal change in various signaling mechanisms, such as photoinduced electron transfer (PET), internal charge transfer (ICT), fluorescence resonance energy transfer (FRET), excimer and so on [30]. There are many reports about a fluorescent sugar sensor based on boronic acids, and they are summarized in some excellent reviews [31–34]. Using hydrogel containing fluorescent boronic acid sensors, Shibata *et al.* have succeeded in continuous monitoring *in vivo* [35,36]. Fluorescent sensors have made a great contribution for the development of the molecular recognition chemistry of boronic acids, and it has a great potential to become alternatives for enzyme-based glucose sensors.

On the other hand, colorimetric sensing systems are suitable for practical use. Although the most widely used glucose meter is based on electrochemistry, there is some commercial, handy-sized blood glucose meters based on colorimetric measurements, which show a performance equivalent to electrochemical sensors [37]. As for urine test strips, we can detect the color change without any devices.

## 4. Colorimetric Sugar Sensor Using Boronic Acid and Azobenzene

The number of colorimetric sugar sensors based on boronic acids is less compared to that of fluorescence sensors [31–34]. The reason for this may be due to the difficulty of the development of a signaling mechanism for the color change of synthetic dyes containing boronic acid.

There has been an interesting approach to produce a color signal without dyes, which uses smart hydrogels that exhibit a change in volume in the response of boronic acid to sugars [38–51]. Sugar-sensitive smart hydrogels with crystalline colloidal arrays or holographic grating reflect light and give a visible narrow band with a wavelength governed by the spacing of the crystal colloid lattice or the holographic fringe. Sugar addition induced a volume change of the smart hydrogels, which results in the shift of the wavelength. These approaches have been summarized in a review [52].

**Figure 2.**
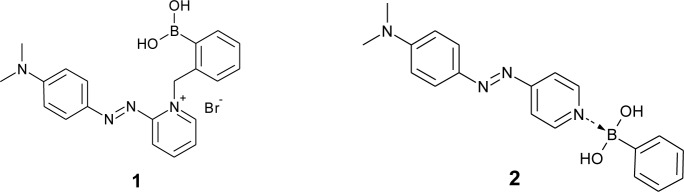
Chemical structures of dye **1** and **2**.

For the development of colorimetric sugar sensors with synthetic dyes, azobenzene has been most widely used. In the 1990s, some boronic acid-appended azobenzene derivatives were synthesized for sugar sensing. Some of them show a sugar response based on a circular dichroism analysis or a change in aggregate formations [53,54]. In 1997, Takeuchi *et al.* developed a conjugate of azo dye and phenylboronic acid (**1**, Figure 2) as a dye that shows an absorption spectral change upon an accompanying structural change [55]. The dye **1** shows an absorption maximum at 558 nm, and the absorption maximum shifted to 568 nm upon the addition of the nucleoside containing *cis*-diol of the ribose moiety. For a clear color change, Koumoto *et al.* used a boron-nitrogen (B–N) interaction between two molecules (**2**, Figure 2) [56]. The original color of the azo dye is yellow in aqueous solution, and the color changed to orange due to the B–N interaction. The color of the solution turned red upon sugar addition.

This is a pioneering work using intermolecular interactions between boronic acid and dyes. The concept of the combination of two molecules has become a growing trend. Many researchers used dyes containing catechol structures, like alizarin red S and pyrocatechol violet [57–60]. These dyes form a cyclic ester with boronic acid, which accompanies a change in the color or fluorescence of the dyes. The sugar addition induced the displacement of dyes from the cyclic ester, which results in a recovery of the original signal of the dyes. These combinations of boronic acids and dyes will offer some interesting applications.

**Figure 3.**
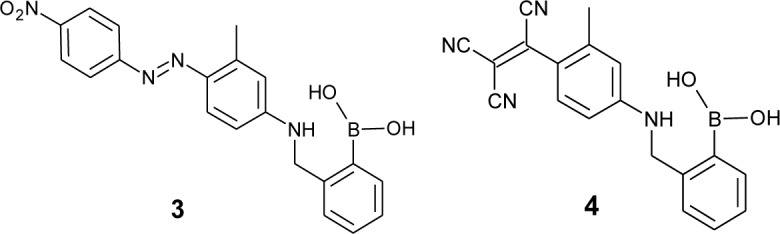
Chemical structures of dye **3** and **4**.

Ideally, a simple system using one constituent would be suitable for practical glucose sensing to avoid interferences in complex biological fluids. In 2000, Ward *et al.* reported a series of dyes with a basic skeleton [61,62]. In particular, dye **3** shows a large color change upon sugar addition in aqueous MeOH at pH 11 (Figure 3). The wavelength shifted *ca*. 55 nm to a shorter wavelength upon sugar complexation, corresponding to the color change from purple to red. They proposed that a key structure of the signaling mechanism is an intramolecular B–N interaction between the boronic acid moiety and the nitrogen of the aniline moiety. They applied the concept to another dye, **4**, which shows a color change in a neutral aqueous solution [63]. In aqueous MeOH at pH 8.2, the addition of sugar induced a shape change in the absorption spectrum, which is recognized as a color change from purple to pink.

**Figure 4.**
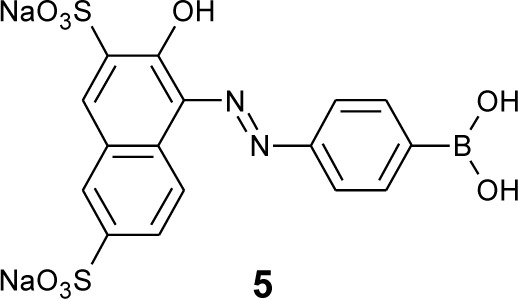
Chemical structure of dye **5**.

DiCesare and Lakowicz have demonstrated that a dye, **5**, works as a visible color sensor in a complete water system at pH 7.0 (Figure 4) [64]. The sugar addition induces a red-shift, which results in a change from orange to a purple-reddish color. They proposed that this color change is due to the conformational change of the boron atom from the neutral sp^2^ form to the anionic sp^3^ form.

## 5. *o*-Boronic Acid Substituted Azobenzene

**Figure 5.**
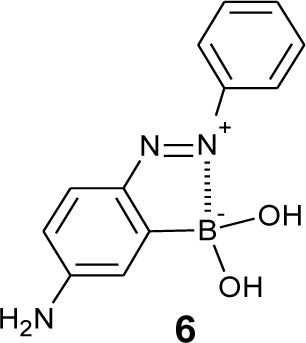
Chemical structure of dye **6**.

**Figure 6.**
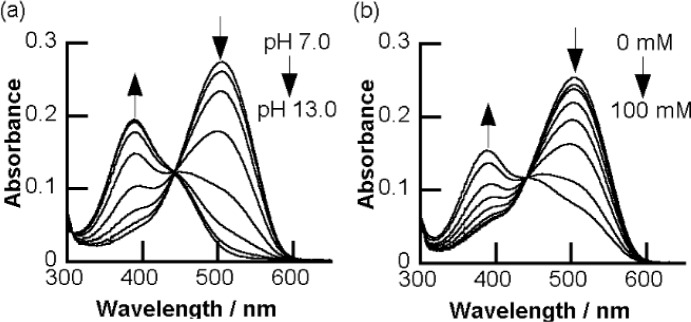
(**a**) UV-visible absorption spectra of dye **6** (10 μM) in different pH solutions (pH 7.0, 10.0, 10.5, 11.0, 11.5, 12.0, 12.5 and 13.0), measured in a methanol/water mixture (1/1, *v*/*v*) containing 4-(2-hydroxyethyl)-1-piperazineethanesulfonic acid (HEPES, 5.0 mM); (**b**) the UV-visible absorption spectra of dye **6** (10 μM) in the presence and absence of D-fructose (0, 1, 2, 5, 10, 20, 50 and 100 mM), measured in a methanol/water mixture (1/1, *v*/*v*) containing *N*-cyclohexyl-2-aminoethanesulfonic acid (CHES, 5.0 mM), pH 10.0. Reprinted with permission from [66]. Copyright 2010 The Chemical Society of Japan.

In order to fabricate sugar sensors that show a significant color change, we have developed a strategy to arrange a boronic acid group adjacent to a chromophore. We expected that a structural change of the boronic acid group upon sugar addition would directly affect the adjacent chromophore. We introduced a boronic acid group to the *o*-position of the azo group. Some *o*-boronic acid substituted azobenzenes were successfully synthesized with diazo-coupling reactions [65,66]. An azo dye, **6** (Figure 5), which has a basic skeleton of a series of *o*-boronic acid substituted azobenzenes, shows an absorption maximum at 505 nm in aqueous MeOH, which is significantly red-shifted compared to that of 4-aminoazobenzene (365 nm) [67]. Figure 6 shows the effect of pH and sugar on the UV-visible absorption spectra of **6**. A pH increase induced a decrease in the absorption maximum at 505 nm and an increase in a new band at 386 nm. Sugar addition induced a similar spectral change. To our knowledge, the dyes containing **6** as a basic skeleton show the largest color change among boronic acid-based sugar sensors. In patents, Russell and Zepp have shown a synthesis of boronic acid azo dyes using a diazo-coupling reaction [68,69]. Although the patents do not contain accurate structures of the dyes, the obtained structure would be similar to **6**.

**Figure 7.**
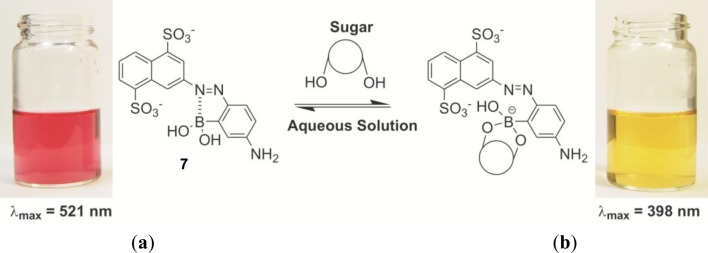
Solutions of dye **7** (20 μM) in CHES buffer (10 mM, pH 10.0), (**a**) in the absence of sugar; and (**b**) in the presence of 100 mM of D-fructose. Reprinted with permission from [65]. Copyright 2007 Elsevier Besloten Vennootschap.

In order to improve the solubility in water, two sulfonyl groups were introduced to the azo dye. The dye, **7**, works as a sugar sensor with completely aqueous system at pH 10, and it showed a drastic changed from red to yellow upon sugar addition (Figure 7), which corresponds to a significant change of the absorption maximum from 521 nm to 398 nm. The binding constants are calculated to be 110 M^−1^ and 6.2 M^−1^ for D-fructose and D-glucose, respectively.

## 6. Investigation of B**–**N Interactions Using ^15^N NMR

**Figure 8.**
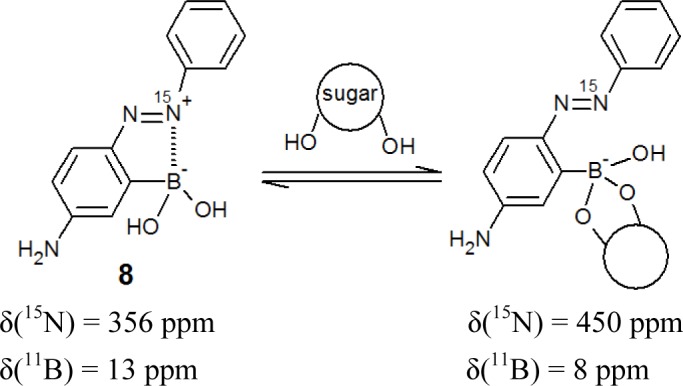
The equilibrium of dye **8** and sugar, and their chemical shifts in multinuclear NMR.

We postulated that the large spectral change of *o*-boronic acid substituted azobenzenes could be explained by B–N interactions between the boronic acid and azo group. In order to gain insight into the B–N interaction, we used ^15^N NMR spectroscopy, because the formations of coordination bonds are sensitively reflected in the ^15^N chemical shifts [70,71]. We synthesized a ^15^N-labelled azo dye (**8**, Figure 8), which corresponds to dye **6** [66].

**Figure 9.**
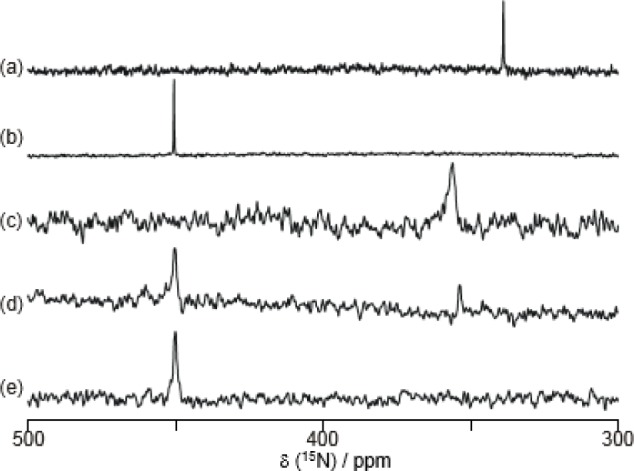
The ^15^N NMR spectra of **8** (20 mM) under various conditions: (**a**) in D_2_O; (**b**) in a 1.0 M NaOD·D_2_O solution; (**c**–**e**) in a mixed solvent (100 mM CHES buffer, pH 10.0/DMSO-*d*_6_ = 3/1, *v*/*v*); (**c**) without D-fructose; (**d**) in the presence of 0.10 M D-fructose; and (**e**) in the presence of 1.0 M D-fructose. The ^15^N-frequency (0 ppm) is 81.07646745 MHz. Reprinted with permission from [66]. Copyright 2010 The Chemical Society of Japan.

Figure 9a shows the ^15^N NMR spectra of **8** in D_2_O. The ^15^N chemical shift was observed at 339 ppm in D_2_O; this value is strongly upfield shifted, because the ^15^N chemical shifts of azo groups are generally observed at around 500 ppm [70]. In contrast, the ^15^N chemical shift of **8** in a 1.0 M NaOD D_2_O solution was a normal value (450 ppm) (Figure 9b).

We used quantum chemical calculations based on density functional theory to optimize the conformation and to predict the value of the ^15^N chemical shift. Based on the value of actual measurements and calculations, we have concluded that dye **8** in neutral solutions has the B–N dative bond, which is responsible for the upfield value of the ^15^N chemical shift around 350 ppm.

Figure 9c–e shows the effect of sugar in the ^15^N NMR spectra of **8**. Sugar addition induced a decrease of the peak at around 350 ppm and an increase of the peak at around 450 ppm. These results demonstrate that adding sugar induces a B–N dative bond cleavage, which results in a recovery of the ^15^N chemical shift in the normal range.

In the UV-visible absorption spectrum (Figure 6), the B–N dative bond causes the significant red-shift of the absorption maximum, and the B–N dative bond is cleaved with sugar addition, which results in a large spectral change.

**Figure 10.**
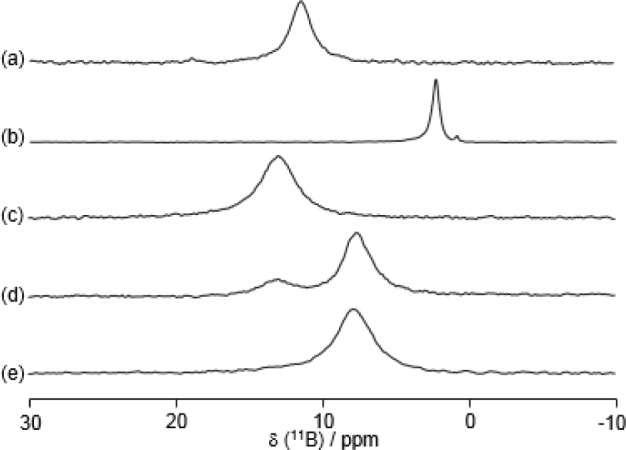
^11^B NMR spectra of **8** (20 mM) under various conditions: (**a**) in D_2_O; (**b**) in 1.0 M NaOD·D_2_O solution; (**c**–**e**) in a mixed solvent (100 mM CHES buffer, pH 10.0/DMSO-*d*_6_ = 3/1, *v*/*v*); (**c**) without D-fructose; (**d**) in the presence of 0.10 M D-fructose; and (**e**) in the presence of 1.0 M D-fructose. The ^11^B-frequency (0 ppm) is Et_2_O·BF_3_ in toluene-*d*_8_. Reprinted with permission from [66]. Copyright 2010 The Chemical Society of Japan.

Boron-11 NMR is a common method to investigate B–N interactions in solution [72–77]. It is known that the ^11^B chemical shifts of sp^3^ and sp^2^ appeared at about zero and 30 ppm, respectively. Figure 10 shows the ^11^B NMR spectra of the same conditions as that for the ^15^N NMR spectra in Figure 9. The addition of D-fructose induced a disappearance of the peak at around 13 ppm and an appearance of the peak at around 8 ppm. Only with this result of ^11^B NMR would it be very difficult to describe the structural change of the B–N motif in **8**, because both the ^11^B chemical shifts at around 13 and 8 ppm correspond to quasi-tetrahedral boron.

We have clearly showed the motion of the B–N motif that plays a key role for the signaling mechanism of *o*-boronic acid-substituted azobenzene. This signaling mechanism corresponds to a solvolysis mechanism, which is proposed by Wang’s group [76,77]. They questioned the signaling mechanism of a fluorescent sugar sensor containing a B–N motif. The B–N motif has been widely used not only in fluorescence sensors, but also in electrochemical and colorimetric sensors [20,21,61–63,78,79]. Previously, ^11^B NMR was the only method to investigate the B–N motif in the dissolved state. Accordingly, it was hard to understand the role of the B–N motif. In fact, the B–N interactions of sugar sensors have been investigated and debated for a long time [72–77]. We have demonstrated that ^15^N NMR will provide a clear picture of the B–N motif, which will contribute to the further development of sugar sensors with the B–N motif.

## 7. Polymers Containing *o*-Boronic Acid-Substituted Azobenzene

**Figure 11.**
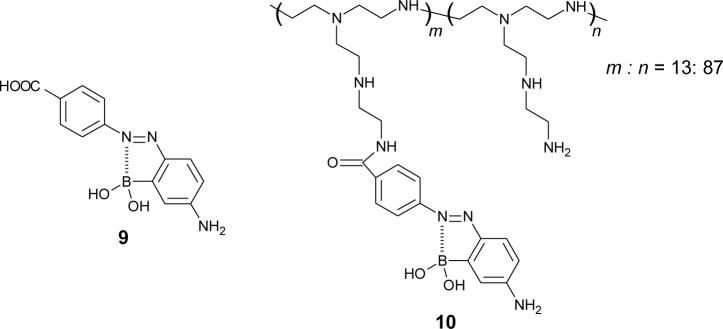
Chemical structures of dye **9** and **10**.

It is known that mono-boronic acid shows a higher affinity for D-fructose compared to D-glucose. Springsteen and Wang calculated the binding constant between phenylboronic acid and sugar. To obtain a higher affinity for D-glucose, we synthesized an azo dye containing carboxylic acid (**9**) and attached it to poly(ethyleneimine) (**10**) (Figure 11) [80], which is a simple and effective strategy for multivalent binding [81,82]. The addition of D-glucose induced a significant change in the UV-visible absorption spectra of polymer dye **10** solutions. In aqueous solution (pH 9.0), the binding constants for D-glucose (*K*_glu_) and D-fructose (*K*_fru_) were calculated to be 54 M^−1^ and 110 M^−1^, respectively (Table 2). The selectivity for D-glucose of dye **10** was increased compared with that of monomeric dye **9** (*K*_glu_ = 1.2 M^−1^, *K*_fru_ = 17 M^−1^).

**Figure 12.**
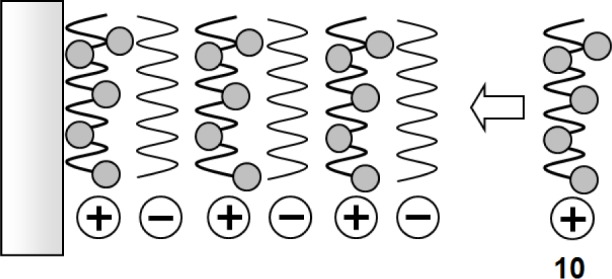
Schematic illustration of fabrication of layer-by-layer films using dye **10**.

Dye **10** has advantage of a good solubility in water, due to the positive charges of the polymer chain. In addition, it can be used in the preparation of multilayered films on the solid surface using layer-by-layer techniques. This technique is based on the alternate adsorption of polyelectrolytes on a solid surface through electrostatic interactions (Figure 12). It is very useful to build functional multilayered films in nanometer-order [83–85]. We fabricated multilayered films composed of dye **10** and polyanions (poly(vinyl sulfate) (PVS), carboxymethylcellulose (CMC)) using the technique. In a (PVS/**10**)_10_ film, a film composed of ten bilayers of PVS and **10**, the affinity for D-glucose was relatively low (*K*_glu_ = 1.7 M^−1^, *K*_fru_ = 28 M^−1^). In contrast, (CMC/**10**)_5_ film, a film composed of five bilayers of CMC and **10**, showed a high affinity for D-glucose (*K*_glu_ = 18 M^−1^, *K*_fru_ = 42 M^−1^). We proposed that the loosely packed structure of the (CMC/**10**)_5_ film probably led to a high affinity for D-glucose [86–88].

**Figure 13.**
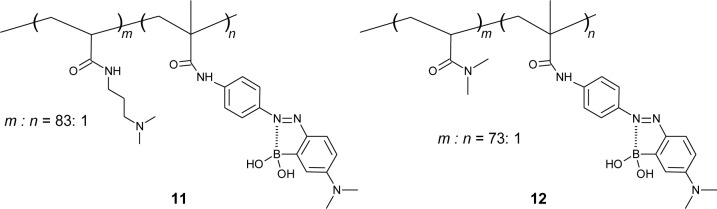
Chemical structures of **11** and **12**.

Okasaka and Kitano synthesized an *o*-boronic acid substituted azobenzene with the vinyl group and use it to prepare copolymers (Figure 13) [89]. They presented the azo polymer containing a tertiary amine in the side chain (**11**), which shows a good sugar response in pH 8.0 solution. On the other hand, the azo polymer containing amide (**12**) shows a sugar response in a more alkaline region (pH > 9). Interestingly, the copolymer showed a change in the fluorescence emission spectra, although azobenzene derivatives usually do not show fluorescence. They proposed that the unordinary fluorescence of the copolymer is derived from the B–N bond, which restricted the photo-isomerization of azobenzene.

## 8. Supramolecular Approaches for Glucose Sensing

**Figure 14.**
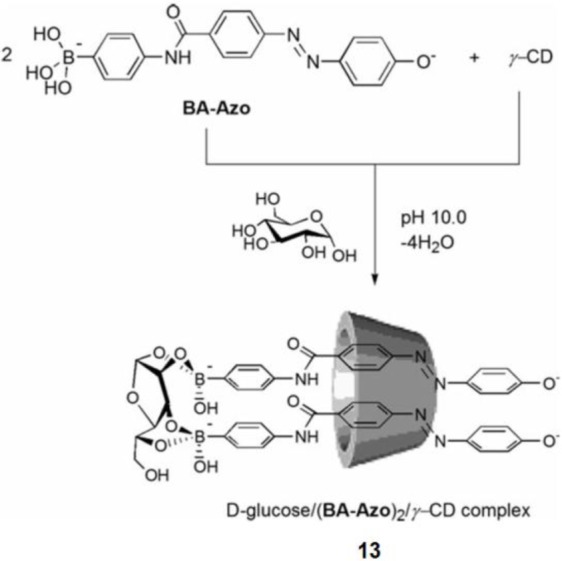
The 2:1 inclusion complex formation of boronic acid-appended azo dye (BA-Azo) with γ-CyD in the presence of D-glucose. Reprinted with permission from [90]. Copyright 2009 The Royal Society of Chemistry.

Shimpuku *et al.* used a unique strategy using supramolecular chemistry to increase D-glucose selectivity [90]. γ-Cyclodextrin (γ-CyD) is a cyclic oligosaccharide composed of eight glucose units, and it works as a host to include guest molecules through hydrophobic interactions. Two molecules of boronic acid-appended azo dye (BA-Azo) are included in the cavity of γ-CyD. In the presence of γ-CyD, BA-Azo exhibits a selective response for D-glucose by forming a dye-D-glucose complex in 2:1 stoichiometry inside the γ-CyD cavity (**13**, Figure 14). They compared some azo dyes and suggested that the difference in the hydrophobic spacer significantly affects the sensing ability for D-glucose.

For a supramolecular approach, we used anionic dye **5** and poly(allylamine) (PAA), which interact through electrostatic force [91]. In pH 7.0 solution, the absorption spectrum of dye **5** was changed upon the addition of PAA, which indicates the formation of aggregates of **5** on the PAA. In the presence of PAA, the binding constant of **5** to D-glucose was improved to 130 M^−1^, which was 93-times higher than the original binding constant, while the binding constant to D-fructose was increased only 3.3-times (Table 3). These results show that the coexistence of PAA can selectively enhance the binding ability of dye **5** to D-glucose. It is expected that two molecules of dye **5** on the surface of PAA cooperatively bind one molecule of D-glucose.

## 9. Other Target Molecules for Boronic Acid Azobenzenes

Human blood glucose level naturally fluctuates throughout the day. In contrast, measuring glycated hemoglobin (HbA_1c_) is useful for assessing the effectiveness of therapy by monitoring long-term serum glucose regulation. HbA_1c_ is a marker for average blood glucose levels over four weeks to three months. As the average concentration of plasma glucose increases, the fraction of HbA_1c_ increases. HbA_1c_ is formed in the Amadori rearrangement, in which glucose and the *N*-end of the β chain form a Schiff base, and it is converted to 1-deoxyfructose. Boronic acids show affinity for this glycated moiety, and boronate affinity chromatographies are practically used for the determination of HbA_1c_ [92–94]. Kim *et al.* have developed a method for the detection of glycated protein on the basis of the spectral shifting of dye **5** [95]. The measurements revealed that the method has a dynamic detection range of 3%–15% (HbA_1c_/total hemoglobin), which covers the required clinical reference range.

**Figure 15.**
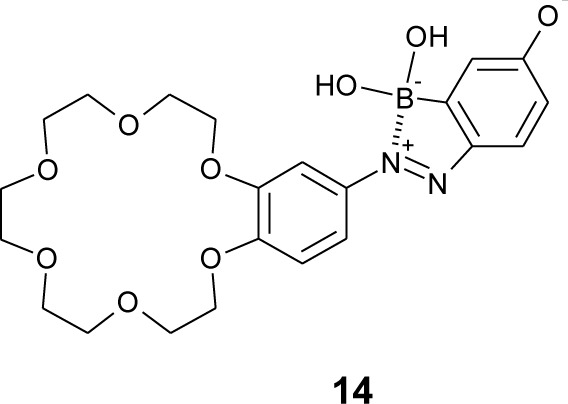
Chemical structure of dye **14**.

Boronic acids have been used to recognize a catechol structure, because its 1,2-diol on the aromatic ring can form a cyclic ester with boronic acids [96–102]. Dopamine is a catecholamine neurotransmitter, and it is associated with several neurological diseases, e.g., Parkinson’s disease and schizophrenia. Monitoring dopamine concentration could be useful for diagnosis and medication treatment for the diseases. Hashimoto *et al.* have designed a boronic acid azo dye (**14**, Figure 15) containing boronic acid and crown ether, which enable two point recognitions through boronate-diol and crown ether-cation interactions [103]. At pH 8, the solution of dye **14** changed its color from red to orange upon the addition of dopamine. The binding constant was 111 M^−1^ in pH 8.0. They showed that dye **14** has a phenolate structure in neutral solution.

## 10. Conclusions

The combination of boronic acid and azo dye has been successfully used for the development of color sensors for a compound containing adjacent diol structure. We developed a series of *o*-boronic acid substituted azobenzenes that shows a drastic color change upon sugar addition. The signaling mechanism was investigated with multinuclear NMR. Especially, ^15^N NMR demonstrated the existence of a B–N dative bond between boronic acid and azo groups well. The B–N dative bond causes a significant red-shift of the absorption maximum, and it is cleaved upon sugar addition, which results in a significant color change. Interestingly, the azo dye containing the B–N dative bond shows fluorescence, which means that the dyes can be a dual colorimetric and fluorescent sensor. The dyes with a B–N dative bond were combined with polymer or crown ether. Polymer appended azo dye **10** shows a high glucose affinity in solutions and in a multilayered film on the surface of a solid. The combination of crown ether and azo dye shows a selective binding ability for dopamine.

The *p*-boronic acid-substituted azobenzenes developed by DiCesare and Lakowicz have an advantage in that they work in a neutral solution, and they were applied for glucose selective binding and HbA_1C_ measurements.

The systems showed in this review have their own advantages, *i.e*., a drastic color change, a high affinity for D-glucose and working in a neutral solution. An ideal colorimetric sensor should include these advantages, and we expect that further study will integrate these advantages. Additionally, it would have great potential to contribute to medical sensors.

## Figures and Tables

**Table 1.** The binding constants of phenylboronic acid to polyol compounds in 0.1 M of phosphate buffer.

Polyol	*K* in pH 6.5/M^−1^	*K* in pH 7.4/M^−1^	*K* in pH 8.5/M^−1^
D-glucose	0.84	4.6	11
D-fructose	29	160	560
sorbitol	47	370	1000
catechol	150	830	3300

**Table 2.** The binding constants of dye **9**, **10** and multilayered films in 10 mM CHES buffer (pH 9.0). PVS, poly(vinyl sulfate); CMC, carboxymethylcellulose.

Dye	*K*_glu_/M^−1^	*K*_fru_/M^−1^	*K*_glu_*/K*_fru_
9	1.2	17	0.071
10	54	110	0.49
(PVS/10)_10_	1.7	28	0.061
(CMC/10)_5_	18	42	0.43

**Table 3.** The binding constants of dye **5** (20 μM) to sugars in the absence and presence of poly(allylamine) (PAA) (10 μg/mL) in 25 mM HEPES buffer (pH 7.0).

Dye	*K*_glu_/M^−1^	*K*_fru_/M^−1^	*K*_glu_*/K*_fru_
5	1.4	75	0.018
5 + PAA	130	250	0.52
